# Peritumoral portal enhancement during transarterial chemoembolization: a potential prognostic factor for patients with hepatocellular carcinoma

**DOI:** 10.1177/02841851211041832

**Published:** 2021-10-19

**Authors:** Sofi Sennefelt Nyman, Angeliki Dimopoulou Creusen, Ulf Johnsson, Fredrik Rorsman, Johan Vessby, Charlotte Ebeling Barbier

**Affiliations:** 1 Department of Surgical Sciences, Section of Radiology, 8097 Uppsala University, Uppsala, Sweden; 2 Department of Medical Sciences, Section of Gastroenterology and Hepatology, 59561 Uppsala University, Uppsala, Sweden

**Keywords:** Hepatocellular carcinoma, transarterial chemoembolization, liver, angiography, abdomen/GI, interventional

## Abstract

**Background:**

Tumor response and survival varies in patients treated with transarterial chemoembolization (TACE) for intermediate stage hepatocellular carcinoma (HCC) and may be associated with several factors.

**Purpose:**

To evaluate safety and efficacy of TACE in patients with intermediate stage HCC and to identify factors related to tumor response and survival.

**Material and Methods:**

Consecutive patients with HCC treated with TACE between September 2008 and September 2018 were retrospectively reviewed.

**Results:**

In 87 patients (71 men; mean age = 68 ± 9 years), 327 TACE treatments were performed (mean = 3/patient; range = 1–12). Mean and median overall survival were 32 and 19 months, respectively. Survival rates at 30 days, one, three, and five years were 99%, 71%, 19%, and 8%, respectively. Objective response (OR) was seen in 84% and disease control (DC) was seen in 92% of the patients. Patients in whom peritumoral portal lipiodol enhancement (PPLE) was seen during TACE had better OR (97 vs. 73%; *P* = 0.007) and DC (100 vs. 85%; *P* = 0.024), and a reduced risk of death (hazard ratio [HR] = 0.52; 95% confidence interval = 0.32–0.86) compared to those without PPLE. Severe adverse events were rare (15%) and occurred more often in patients with a larger tumor size.

**Conclusions:**

TACE was effective and safe in patients with intermediate stage HCC. Patients with PPLE during TACE had better tumor response and longer survival than those without PPLE. Severe adverse events occurred more often in patients with larger tumors.

## Introduction

Hepatocellular carcinoma (HCC) is the fourth most common cause of cancer-related death in the world, representing the seventh most common cancer (1). The incidence of HCC is expected to increase (2). HCC occurs mainly in patients with chronic liver diseases, such as cirrhosis caused by viral hepatitis, alcohol fatty liver disease (AFLD), and non-alcoholic fatty liver disease (NAFLD) (3–5).

The recommended treatment of intermediate stage HCC is transarterial chemoembolization (TACE), conventional or with drug eluting beads (DEB-TACE) (6), since it prolongs survival (7). No major difference in efficacy and safety has been found between conventional and DEB-TACE (8,9). Local variations between treatment centers occur concerning the conventional TACE procedure. The addition of an embolic agent at the end of the procedure has not been proved to influence overall survival or tumor response (10,11).

Adverse events (AE) related to TACE are usually transient and treatable (12). Severe adverse events (SAE) are associated with advanced liver disease (Child-Pugh B and C), a central tumor localization, and a large tumor volume (13).

The efficacy of TACE varies concerning tumor response and survival (8,13–17) and may be associated with several factors such as tumor size, Child Pugh class, Barcelona clinic liver cancer (BCLC) stage, tumoral enhancement or peritumoral portal lipiodol enhancement (PPLE) during TACE, serum C-reactive protein (CRP), serum albumin, and alpha-fetoprotein (AFP) levels (18–25).

The aim of the present study was to evaluate the safety and efficacy of TACE and to identify factors related to tumor response and survival in patients with intermediate stage HCC. A hypothesis was that arterial tumor enhancement on pre-TACE CT, tumor enhancement during TACE, and PPLE would reflect tumor response and survival.

## Material and Methods

### Patient data

All patients with HCC who had their first TACE treatment at our University Hospital between September 2008 and January 2018 were identified. A retrospective review was performed after approval from the regional research ethics and review board. Informed consent was sent to patients who were still alive (n = 16) for their signature. Data concerning AEs, biomarkers, previous treatments, presence and etiology of cirrhosis, clinical ascites, body mass index (BMI), and date of death were obtained from medical records (SSN). The diagnosis of cirrhosis was determined according to clinical routine based on biopsy, imaging, clinical, anamnestic findings, and laboratory parameters.

The picture archiving system and the radiology information system were reviewed concerning the TACE procedures, magnetic resonance imaging (MRI), and computed tomography (CT) examinations (SSN, CEB).

The Common Terminology Criteria for Adverse Events (CTCAE) version 4 (26) was used to categorize AEs occurring within 30 days. Post-embolization syndrome (PES) was defined as one or several of the following symptoms: pain; fever; nausea; and vomiting. The cause of death was retrieved from the national cause of death register at the national board of health and welfare.

### TACE procedure

The TACE procedure was performed by an interventional radiologist (CEB, ADC, or UJ) in a standardized manner. To confirm patent portal vein circulation before treatment, either ultrasound or prolonged angiographic imaging was used. The femoral artery was punctured under local anesthesia. Selective angiography of the superior mesenteric artery and the coeliac trunk was performed in order to determine the arterial anatomy, detect accessory tumor feeders, and arterioportal shunts. The latter was considered a contraindication to TACE.

The right or left hepatic artery feeding the tumor or tumors was selectively catheterized and embolized with an emulsion of 50 mg of doxorubicin (Adriamycin; Pfizer Inc., New York, NY, USA) mixed with 13 ml of Lipiodol Ultra Fluid (Guerbet, Aulnay-sous-Bois, France) until the total amount was given or until arterial stasis was achieved. A lobar catheter position was chosen to attain treatment to potential accessory tumors. No embolic material was added since frequent repeated treatments were planned. If there were tumors in both liver lobes, the treatment sessions were altered between the lobes, beginning with the lobe with the largest tumor burden. If the treatment was tolerated by the patient, it was repeated every 6–8 weeks and an evaluation with CT or MRI was performed after treatment 3 or 5. If viability was still seen, treatments were pursued with the same intervals until complete response or progressive disease as long as no major adverse effects occurred.

### Tumor evaluation

**Fig. 1.**
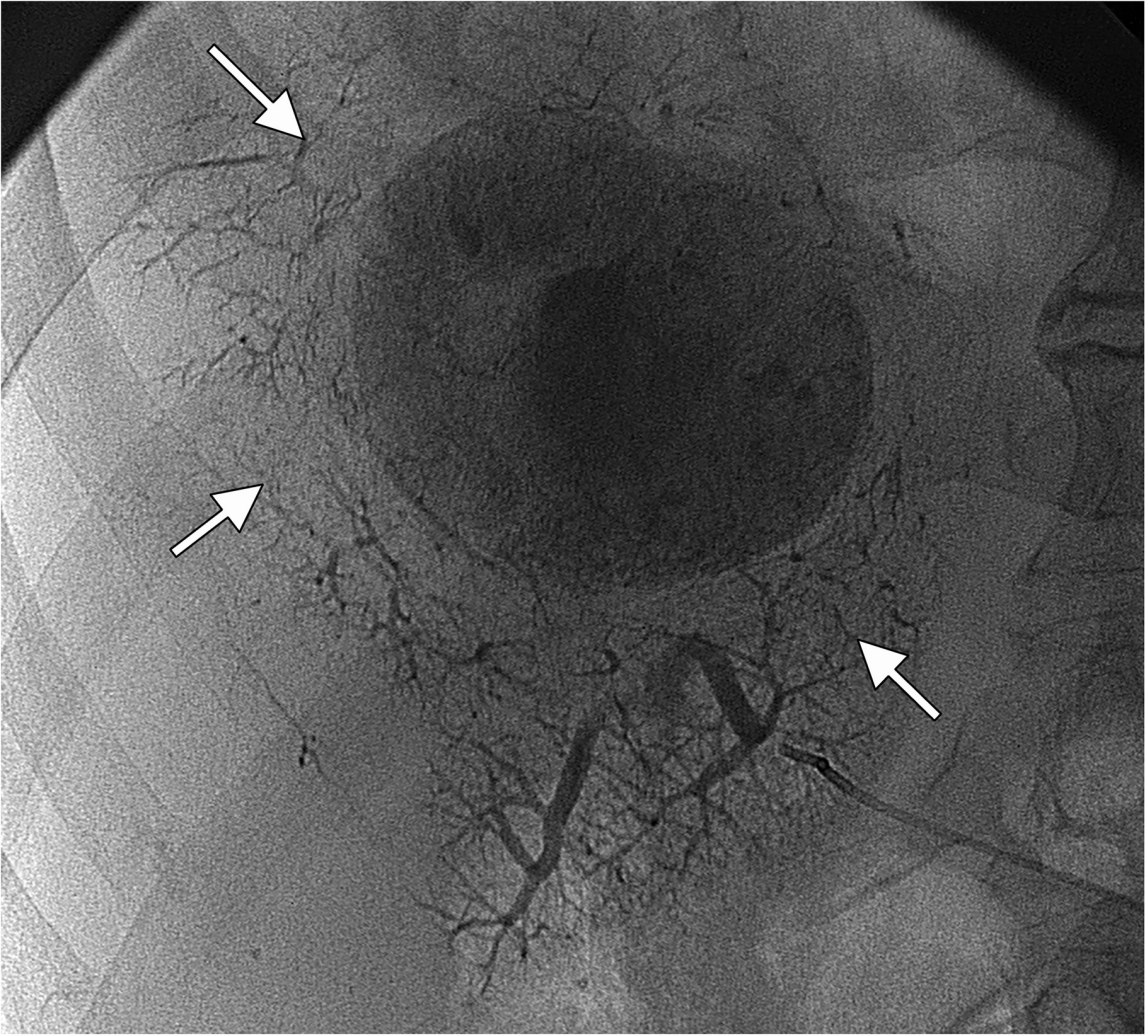
Radiographic image displaying PPLE (arrows) during TACE for HCC. In this 86-year-old man, pathological anatomical diagnosis had demonstrated a highly differentiated tumor. Complete remission was achieved, and the patient was still alive at follow-up after eight years. HCC, hepatocellular carcinoma; PPLE, peritumoral portal lipiodol enhancement; TACE, transarterial chemoembolization.

To evaluate tumor enhancement a region of interest was drawn on the slice where the tumor appeared largest and most enhanced on arterial phase contrast-enhanced CT before TACE. Tumor lipiodol enhancement was graded from no enhancement to dense enhancement (0–2) on TACE images. PPLE was defined as lipiodol persisting in peritumoral portal vessels during TACE (example displayed in Fig. 1).

To evaluate tumor response, MRI and CT images that had been obtained as close as possible before TACE (mean = 10 weeks) were compared to the images obtained as close as possible after the last TACE treatment (mean = 9 weeks). Measurements were performed on the three largest tumors (SSN) in the treated lobe/lobes, according to the modified Response Evaluation Criteria in Solid Tumors (mRECIST) (27), considering a decrease of viable tumor diameter ≤30% as response and an increase ≥20%, or the appearance of a new lesion in the treated lobe, or of extrahepatic tumor, as progress. In patients where there was no post-TACE CT or MRI, assessments were performed on ultrasound (n = 3). Thus, the accumulative response was evaluated for each patient after all TACE treatments had been performed. Objective response (OR) was defined as complete remission (CR) plus partial response (PR). Disease control (DC) was defined as OR plus stable disease (SD).

### Statistical analysis

SPSS Statistics version 23 (IBM Corp., Armonk, NY, UA) was used for statistical analyses. The T-test with Levene’s test for equal variances was used to compare means between groups to evaluate tumor response, treatment effect on biomarkers, and to compare the severity of AEs and the effect on biomarkers. Continuous parameters were analyzed using Spearman rank correlation and categorical parameters were analyzed the using Pearson chi-square test. The Cox proportional hazards model and the Kaplan–Meier method were used to estimate survival, when the observed variable was categorical, starting from the day of the first TACE treatment. Simple and multiple regression analysis was used to analyze factors associated to survival and tumor response. The Mann–Whitney test was used to compare the groups with and without PPLE. The significance level was set at *P* < 0.05 in all analyses.

## Results

### Demographics

Between September 2008 and September 2018, 89 consecutive patients with HCC were treated with TACE. Two patients were excluded from the study: one due to emigration and the other because no written informed consent was received. The remaining 87 patients constituted the study population of the present study. The mean follow-up time was 26 months (range = 5 days–126 months). A total of 327 TACE treatments were performed (median 3/patient; range = 1–12).

In 5 of 327 treatments where lipiodol and doxorubicin were not available at the pharmacy, DC Beads (Terumo Corp., Tokyo, Japan) filled with 150 mg of doxorubicin was used. The mean administrated doses of doxorubicin at the first five TACE treatments were 49 mg, 43 mg, 40 mg, 22 mg, and 12 mg, respectively, and the mean total dose was 160 ± 109 mg.

Baseline characteristics are presented in Table 1.

**Table 1.** Baseline characteristics of 87 patients with HCC treated with TACE.

Characteristics	Value
Sex (F)	16 (18)
Age (years)	68 ± 9 (45–88)
BMI	28 (17-42)
Clinical liver cirrhosis	68 (78)
Viral hepatitis*	13 (15)
Alcoholic fatty liver disease	12 (14)
Alcoholic fatty liver disease + viral hepatitis^†^	20 (23)
Non-alcoholic fatty liver disease	6 (7)
Others^†^	17 (19)
No clinical liver cirrhosis	9 (10)
HCC diagnosed with biopsy, AFP + imaging, no information	6, 2, 1
No information on liver cirrhosis	10 (12)
HCC diagnosed with biopsy, AFP + imaging, no information	3, 4, 3
BCLC (A (%)/B (%)/C (%))	29 (33)/49 (56)/9 (11)
Child Pugh (A (%)/B (%))	77 (88)/10 (12)
Systemic chemotherapy and/or radiation pre-TACE	7 (8)
Surgical resection and/or ablation pre-TACE	13 (15)
Number of tumors	3.4 (1–50)
1	36 (41)
2	19 (22)
>2	31 (37)
Bilobar	30 (35)
Largest tumor diameter (cm)	6.2 (1.6–19)
Transplanted after TACE	4 (5)
AFP^‡^	941 (2–14,480)

Values are given as n (%), mean ± SD (range), or mean (range) unless otherwise indicated.

*Viral hepatitis B or C.

^†^ Others include: alcoholic fatty liver disease + with non-alcoholic fatty liver disease (n = 3); hemochromatosis (n = 1); porphyria (n = 2); hemochromatosis + alcohol fatty liver disease + viral hepatitis (n = 1); hemochromatosis + alcoholic fatty liver disease + non-alcoholic fatty liver disease (n = 1); and unclear etiology (n = 9).

^‡^ Only available in 81 patients.

AFP, alpha-fetoprotein; BCLC, Barcelona Clinic Liver Cancer; BMI, body mass index; HCC, hepatocellular carcinoma; TACE, transarterial chemoembolization.

### Tumor response

There were no post-TACE images in 14 of the patients. Thus, 73 patients were included in the mRECIST assessments (Table 2). OR was seen in 84% (n = 61/73) and DC was seen in 92% (n = 67/73) of patients.

**Table 2.** Accumulative tumor response, for the entire study period, using mRECIST in 73 patients with HCC who had TACE.

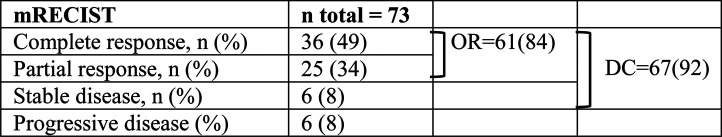

Values are given as n (%) unless otherwise indicated.

DC, disease control; HCC, hepatocellular carcinoma; mRECIST, modified Response Evaluation Criteria in Solid Tumors; OR, objective response; TACE, transarterial chemoembolization.

OR or DC was more frequent in patients in whom PPLE was achieved during any of the TACE procedures compared to in those in whom PPLE was never achieved (97 vs. 73%; *P* = 0.007 and 100 vs. 85%; *P* = 0.024, respectively). There was also a positive correlation between the number of times that PPLE occurred in a patient (including the first five TACE treatments) and the occurrence of OR (n = 31/61 vs. 1/12; *P* = 0.012; correlation coefficient = 0.29) or DC (n = 32/67 vs. 0/6; *P* = 0.031; correlation coefficient = 0.25). Objective response was more frequently obtained in patients with (n = 21/21) than patients without (n = 39/51) PPLE during first TACE (*P* = 0.015). In the simple and multiple regression analyses, PPLE was associated with OR (*P* = 0.009 and 0.049, respectively). Factors analyzed in the regression analysis are displayed in Table 3. There was no difference between the groups with or without PPLE regarding age, sex, size of the largest tumor, whether the tumors were uni- or bilobar, Child Pugh class, BCLC stage, AFP < 400 μg/L, CRP < 10 mg/L, or albumin <35 g/L. In 68 patients, where image quality allowed quantification of the necrotic part of the largest tumor, the necrotic part was greater in patients with PPLE (n = 30) compared to those without PPLE (n = 38) (mean = 85% and 67%, respectively; *P* = 0.026).

**Table 3.** Simple and multiple regression analysis of factors associated with OR in 73 patients with HCC treated with TACE (patients were divided into two groups: responders and non-responders to TACE).

Parameters	Responders*	Non-responders*	*P* value simple analysis	*P* value multiple analysis
Mean age (years)	67	68	0.630	0.535
Sex (F)	12/61 (20)	1/12 (8)	0.330	0.177
PPLE	31/61 (51)	1/12 (8)	0.009	0.049
Largest tumor >8 cm	10/61 (16)	2/12 (17)	0.989	0.381
Single tumors compared to multiple	25/61 (41)	2/12 (17)	0.089	0.196
Unilobar tumors compared to bilobar	42/61 (69)	6/12 (50)	0.474	0.797
AFP >400 μg/L	53/56 (95)	9/11 (82)	0.182	0.171
Albumin <35 g/L	33/59 (56)	9/12 (75)	0.140	0.346
CRP < 10 mg/L	42/56 (75)	9/11 (82)	0.586	0.400

Values are given as n (%) unless otherwise indicated.

*Responders = OR according to mRECIST.

AFP, alpha-fetoprotein; CRP, C-reactive protein; HCC, hepatocellular carcinoma; mRECIST, modified Response Evaluation Criteria in Solid Tumors; OR, objective response; PPLE, peritumoral portal lipiodol enhancement; TACE, transarterial chemoembolization.

There was no association between tumor response and the arterial enhancement on pre-TACE CT, the occurrence of tumor lipiodol enhancement during TACE, the total dose of doxorubicin, tumor size (>8 cm in 12/73 patients), type of cirrhosis, serum levels of albumin, CRP, and AFP before the first treatment.

### Survival

Mean overall survival was 32 months (95% confidence interval [CI] = 24–40) and median 19 months (range = 0–126 months), evaluated in 83 of the patients (excluding four patients who had a liver transplantation after TACE). Survival rates at 30 days, one, three, and five years were 98%, 71%, 20%, and 8%, respectively. At the time of the analysis, 84% (n = 70/83) of the patients were deceased. In 83% (n = 57/69) of these patients, the cause of death was related to cirrhosis and HCC.

**Fig. 2.**
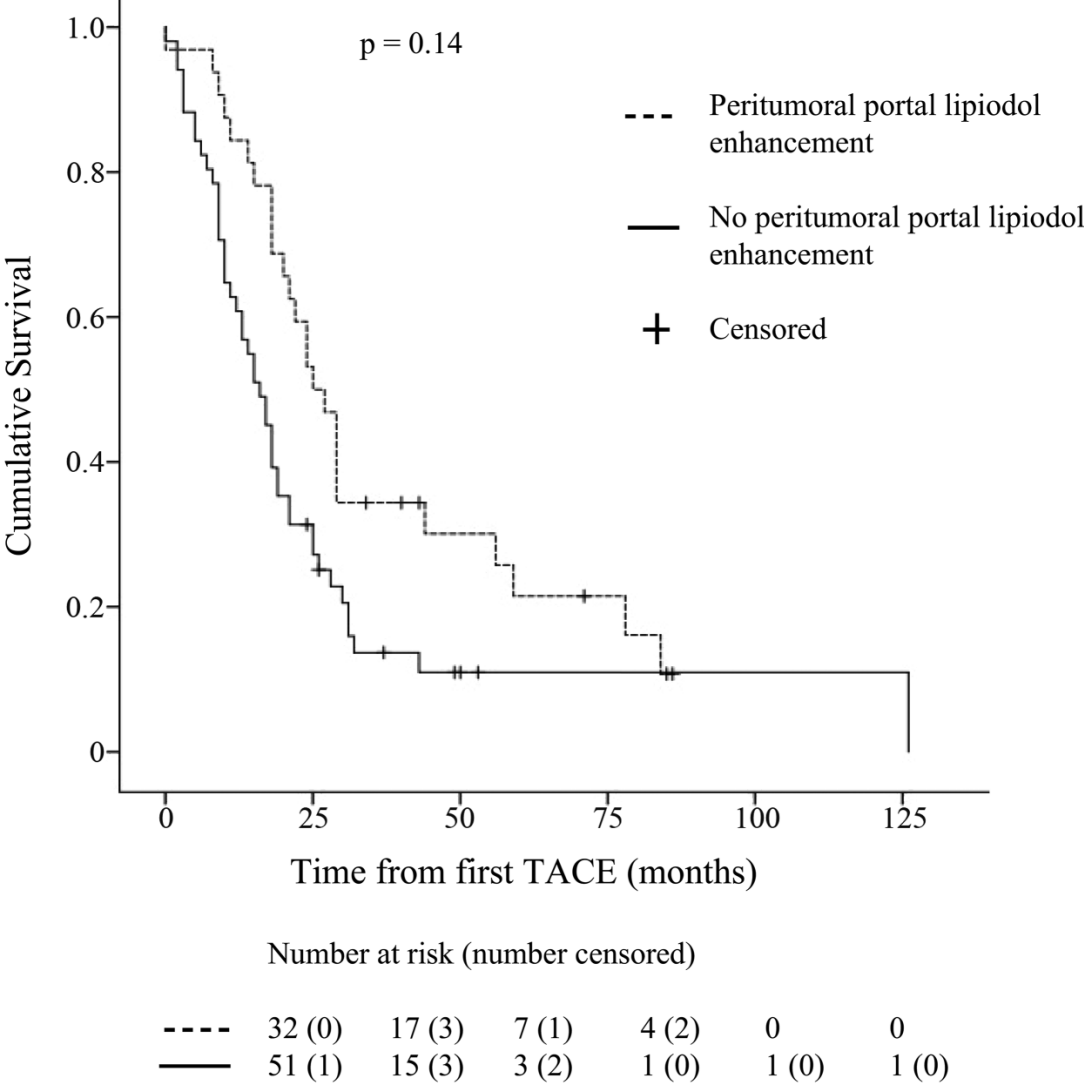
Kaplan–Meier graph displaying survival in 83 patients with HCC with or without PPLE during TACE. HCC, hepatocellular carcinoma; PPLE, peritumoral portal lipiodol enhancement; TACE, transarterial chemoembolization.

**Fig. 3.**
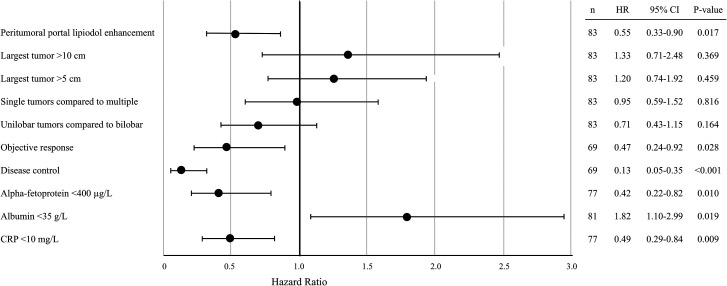
Forest plot displaying hazard ratios for death in main analysis parameters in patients with HCC treated with TACE. Objective response and disease control were calculated according to the mRECIST criteria for tumor response. CRP, C-reactive protein; HCC, hepatocellular carcinoma; mRECIST, modified Response and Evaluation Criteria in Solid Tumors; PPLE, peritumoral portal lipiodol enhancement; TACE, transarterial chemoembolization.

Patients with PPLE survived longer than those without PPLE (mean = 37 months; 95% CI = 28–47 and 28 months; 95% CI = 17–38, respectively; *P* = 0.014) (Fig. 2). Hazard ratios for death are displayed in Fig. 3. Survival >24 months was associated with PPLE in both the simple and the multiple regression analyses (*P* = 0.012 and 0.017, respectively). Factors analyzed in the regression analysis are displayed in Table 4. Child Pugh class, the BCLC classification, or the etiology of the patient's liver cirrhosis did not influence the risk of death. There was no association between survival and the total amount of doxorubicin that a patient received.

**Table 4.** Simple and multiple regression analysis of factors associated with survival in 82 patients with HCC treated with TACE (patients were divided into two groups: survival 24 months or shorter, and survival longer than 24 months).

Parameters	Survival ≤24 months	Survival >24 months	*P* value simple analysis	*P* value multiple analysis
Mean age (years)	68	68	0.740	0.571
Sex (F)	12/51 (24)	4/32 (13)	0.511	0.595
PPLE	15/51 (29)	17/32 (53)	0.012	0.017
First PPLE treatment*	10/51 (20)	10/32 (31)	0.056	-
Largest tumor >8 cm	11/51 (22)	7/32 (22)	0.882	0.188
Single tumors compared to multiple	24/51 (47)	11/32 (34)	0.938	0.286
Unilobar tumors compared to bilobar	33/51 (65)	23/32 (72)	0.263	0.172
AFP >400 μg/L	40/49 (82)	26/28 (93)	0.233	0.195
Albumin < 35 g/L	35/50 (70)	14/31 (45)	0.250	0.783
CRP < 10 mg/L	33/50 (66)	22/27 (81)	0.089	0.084

Values are given as n (%) unless otherwise indicated.

*First PPLE treatment was not included in the multivariate analysis.

AFP, alpha-fetoprotein; CRP, C-reactive protein; HCC, hepatocellular carcinoma; mRECIST, modified Response Evaluation Criteria in Solid Tumors; OR, objective response; PPLE, peritumoral portal lipiodol enhancement; TACE, transarterial chemoembolization.

The size of the largest tumor in these 83 patients was >5 cm in 46, >8 cm in 18, and >10 cm in 13 patients.

### Adverse events and biomarkers

Of the patients, 61%–70% had no perioperative AEs during the first five treatments (Table 5) and in 42% (n = 137/327) of the TACE treatments no postoperative AEs occurred. The frequency of post-TACE PES is presented in Table 6.

**Table 5.** Adverse events in patients with HCC during TACE treatment, observed at the interventional radiology department.*

During TACE	TACE 1 (n = 85)	TACE 2 (n = 68)	TACE 3 (n = 57)	TACE 4 (n = 40)	TACE 5 (n = 25)
No complication	52 (61)	46 (68)	39 (68)	28 (70)	17 (68)
Nausea/Pain	28 (33)	22 (32)	17 (30)	12 (30)	8 (32)
Hypotension	6 (7)	0	2 (4)	2 (5)	0
Bleeding/Hematoma	4 (5)	0	0	0	0

Values are given as n (%).

*First five treatments observed. Patients could have more than one complication.

HCC, hepatocellular carcinoma; TACE, transarterial chemoembolization.

**Table 6.** Patients with HCC experiencing PES* after TACE.

TACE treatment	Post-TACE PES
TACE 1	56/87 (65)
TACE 2	43/68 (64)
TACE 3	29/57 (51)
TACE 4	18/40 (45)
TACE 5	12/25 (48)

Values are given as n (%).

*Defined as one or several of the following symptoms: pain; fever; nausea; and vomiting.

HCC, hepatocellular carcinoma; PES, post-embolization syndrome; TACE, transarterial chemoembolization.

Mild postoperative AEs (CTCAE 1–2) occurred after 43% of the treatments (n = 142/327) and SAEs (CTCAE 3–5) occurred after 15% (n = 48/327) of the treatments (detailed information is presented in Supplementary Table 1). The risk of experiencing a SAE increased with increasing size of the largest tumor (*P* = 0.003). Patients with life-threatening AEs within 30 days (CTCAE grades 4 and 5) are further described in Supplementary Table 2.

Alanine transaminase, aspartate aminotransferase, bilirubin, and international normalized ratio (INR) increased slightly after each TACE compared to levels before first TACE (*P* < 0.05). All but bilirubin were normalized when controlled after the last treatment. This was not considered an AE. However, there was a positive association between these biomarkers after TACE and the CTCAE grade after first treatment (*P* = 0.017, 0.010, 0.033, and 0.028, respectively).

Four patients received only one TACE, despite having no postoperative AE, due to progression of liver cirrhosis (n = 1), tumoral progression (n = 1), vasovagal reaction during the second TACE disabling finalization, and due to severe abdominal pain and hypotension during the first treatment (n = 1).

## Discussion

There was good tumor response to TACE in the present study (OR = 84%, DC = 92%), consistent with other cohorts (OR = 54%–90%, DC = 85%–94%) (8,14,16). Overall mean and median survival were in the higher range (32 vs. 19 months) compared to other reports (mean = 23–31 months; median = 16–19 months) (13,15,17). Survival at one, three, and five years was consistent with other cohorts (i.e. 71%, 19%, and 8%, respectively, compared to survival at one, three, and five years of 70%, 40%, and 32%, respectively in a large meta-analysis (13) survival at one and three years of 46% and 8% in another (28)), indicating that TACE improves short-term survival, but does not necessarily influence long-term survival. The three- and five-year survival rates in the present study are limited by the mean follow-up time being only 26 months.

In the present study, the risk of death was reduced by half in patients with PPLE during any of the TACE treatments, whereas PPLE during the first TACE was not associated to survival (*P* = 0.056). This might suggest that a patient well enough to receive several treatments will have an increased chance of PPLE. However, our observation that PPLE during any of the TACE treatments reduced the risk of death confirms that not obtaining PPLE during first TACE must not indicate a bad prognosis. Thus, not having obtained PPLE during first TACE should not discourage from further TACE treatments. Perhaps some tumors need more than one treatment before the emulsion fills up the tumor and reaches the portal vessels. Therefore, having obtained PPLE during TACE might be considered a predictor of increased tumor response and survival compared to not having obtained PPLE. To the best of our knowledge, an association between PPLE and survival has not been reported before.

Our observation that PPLE during first TACE and during any TACE were independently positively correlated to tumor response supports the results of previous studies where the local recurrence rate of HCC is lower in patients with intense PPLE (18,29). It has been suggested that lipiodol would more easily pass through a moderately than a poorly differentiated tumor and thereby provide a greater ischemic effect, since there is a more complete outflow tract (30). This might be supported by the observation in one of our patients, displayed in Fig. 1. Therefore, PPLE could indicate the extent to which the tumor has been permeated with the lipiodol emulsion and, thus, exposed to the drug. Furthermore, a correlation between PPLE intensity and the extent of histopathological necrosis has been described (31), which may provide a pathophysiological explanation to the positive association between PPLE and tumor response and survival in the present study. However, a potential association between tumor differentiation and PPLE remains to be explored.

In a retrospective study of 105 patients, who received TACE as a bridge to transplantation, intense tumor enhancement during TACE was positively correlated to tumor response (19). In the present study, no such association was found. This might be explained by the fact that the tumors in the present study were generally larger with a larger distribution volume for the lipiodol and a larger area of tumor necrosis diluting the enhancement and making it less intense. In some studies, a correlation has been observed between a larger tumor size and a poorer tumor response and survival (20,23,25,32,33), whereas no such correlation has been found in other studies (34,35). Even though there was a significant variation of tumor size in the present study, with 22% (n = 18/82) of the tumors being >8 cm in survival calculations and 16% (n = 12/73) in the tumor response calculations, there were no associations between tumor size and response or survival. This might partly be explained by the fact that the studies reporting a correlation between tumor size and outcome were performed in cohorts with a generally poorer liver status than our cohort. Thus, the decision to perform TACE should not exclusively be determined by the size of the tumor. However, increased attention to AE may be needed since an association was observed between SAE and a large tumor size (*P* = 0.003).

Elevated levels of CRP (21) and AFP (22), and decreased levels of albumin (25) are all predictors of poor outcome, which is confirmed by the results of the present study.

Lower Child Pugh class and BCLC stage before first treatment have been found to positively affect survival rates (23,24). This was not found in our cohort, perhaps due to a rather homogenic study population (9/87 BCLC C and 10/87 child Pugh B).

Only 2% of the TACE treatments (n = 7/327) entailed life-threatening AEs. Some of these could have been avoided: the symptoms of a myocardial infarction were misinterpreted as PES in one patient; TACE was performed despite imaging signs of a liver abscess in another; and in a patient with unrecognized active alcohol abuse. The increase in liver enzymes, bilirubin, and INR after TACE, in the present study, was not considered an AE since a transient elevation is expected (13).

The present study has some limitations. It was limited by its retrospective design and by the fact that the follow-up time was short in some patients. The study population is rather homogenic with most of the patients having a well-preserved liver function. When assessing tumor response and AEs, there is room for subjectivity by the interpreter. In order to minimize this, categorization was performed using validated instruments such as mRECIST and CTCAE.

In conclusion, TACE was effective and safe in patients with intermediate stage HCC and relatively preserved liver function. Patients with PPLE during TACE had better tumor response and longer survival than those without PPLE. Tumor size or tumor lipiodol enhancement during TACE was not associated to tumor response or survival. However, SAEs occurred more often in patients with larger tumors compared to in those with smaller tumors.
